# Inhibiting IL-17A and IL-17F in Rheumatic Disease: Therapeutics Help to Elucidate Disease Mechanisms

**DOI:** 10.1007/s11926-022-01084-4

**Published:** 2022-07-21

**Authors:** Hoi Ki Joshua Tam, Philip C. Robinson, Peter Nash

**Affiliations:** 1 Ipswich Public Hospital, Queensland Health, Ipswich, QLD Australia; 2 The University of Queensland, Herston, QLD 4006 Australia; 3 Department of Rheumatology, Royal Brisbane & Women’s Hospital, Herston, QLD Australia; 4 School of Medicine and Dentistry, Griffith University, Gold Coast, QLD Australia

**Keywords:** IL-17, Ankylosing spondylitis, Psoriatic arthritis, Secukinumab, Ixekizumab, Brodalumab, Bimekizumab

## Abstract

**Purpose of Review:**

Psoriatic arthritis and ankylosing spondylitis belong to a family of rheumatological diseases that lead to painful joint inflammation that impacts on patient function and quality of life. Recent studies have shown that the pro-inflammatory cytokine IL-17 is involved in the inflammatory joint changes in spondyloarthritides. We will review the pathophysiology of IL-17 and review the biological therapies targeting IL-17.

**Recent Findings:**

IL-17 is produced and released from T cells and is dependent on multiple upstream cytokines, which include IL-23. There are six members of the IL-17 family that are secreted from multiple populations of T cells. The initial biologic medications have been developed against IL-17A, which is the best-studied member of this family. These medications appear to be effective in controlling joint inflammation, improving patient quality of life, and are generally well tolerated. More recently, medications have been developed that target both IL-17A and IL-17F. In addition, brodalumab, an antibody targeting the IL-17 receptor, has had a resurgence after initial concerns for an increased risk of suicide.

**Summary:**

IL-17 is an inflammatory cytokine that is critical in the pathobiology of axial spondyloarthritides. Recent biological therapies targeting IL-17A are effective and well tolerated in patients with axial spondyloarthritis. Specific targeting of the Il-17A/F heterodimer is also effective and provides another viable option in the clinician’s armamentarium.

## Introduction to IL-17

Interleukin (IL)-17 is a family of pro-inflammatory cytokines, of which there are six known members (IL-17 A–F). IL-17A is the founding, and best-studied, member of the family, and was initially detected in murine lymphoid cells [1]. The functions of the IL-17 cytokines are varied and complex. While many IL-17 family members are pro-inflammatory and act in pathogen defence and immune-mediated disease, they also have roles in mucosal integrity, allergen response, anti-inflammation, and regulation of lymphocyte function. They can act in an autocrine manner and can influence the function of other cytokines, including other Il-17 family members. These IL-17 cytokine functions are briefly summarized in Table 1 and are fully reviewed by Zhang and colleagues [2].

**Table 1:** Summary of IL-17 family members and their cognate receptors

Family member	Functions	Receptor
IL-17A	•Protection against bacterial and fungal infection •Pro-inflammatory function in immune-mediated diseases •Regulation of Th17 cell function and migration •Mucosal integrity	IL-17RA, IL-17RC, IL-17RD
IL-17B	•Immune-mediated arthritis in mouse model of disease •Anti-inflammatory role in the colon antagonizing the gut-inflammatory action of Il-17E	IL-17RB
IL-17C	•Inflammatory response to infection and immune-mediated disease •Upregulated early as compared to IL-17A •Autocrine potentiation of Th17 cell response	IL-17A, IL-17-RE
IL-17D	•Increased production of inflammatory cytokines in endothelial cells •Protective effect in experimental colitis in mice •Promotes natural killer cell targeting of tumour cells	CD93
IL-17E	•Promote the allergic response in Th2 and mast cells	IL-17RA, IL-17RB
IL-17F	•Similar to IL-17A signalling, can form heterodimers with IL-17A and bind to common receptors	IL-17RA, IL-17RC, Il-17-RD

The functions of IL-17A and IL-17F are the best understood, and as such are the main therapeutic targets. IL-17A and IL-17F can form homo- and heterodimers that bind to the IL-17 receptor to activate downstream signalling pathways leading to the release of pro-inflammatory cytokines [2–9]. The IL-17 receptor is formed by homo- or heterodimers of IL-17 receptor A and IL-17 receptor C (IL-17RA and IL-17RC, respectively). IL-17 receptor activation leads to canonical signalling and gene expression via the NF-κB, MAP kinase, and CCAAT/enhancer binding protein-B signalling pathways [10, 11••]. Activated IL-17 receptors can also interact with other cell surface receptors (EGF receptor, FGF receptor, NOTCH1, and C-type lectin receptor) to promote cell proliferation and tissue repair [12]. Functionally, IL-17 is involved in the body’s response to bacterial and fungal infection. IL-17 has been shown to be involved in fracture repair and osteogenesis [13, 14]. Indeed, the anatomical sites affected in axial spondylarthritis (axSpA) are sites of repeated micro-trauma [15]. However, dysregulation of IL-17 can lead to autoimmune disease [16, 17].

IL-17 is released from several cells, including Th17 helper cells, γ/δ T cells, interstitial lymphoid cells, neutrophils, and mast cells [18•, 19, 20]. The major source of IL-17 appears to be Th17 cells [21, 22]. The production and secretion of IL-17 cells appears to be dependent on the IL-23 cytokine, a cytokine released from antigen-presenting cells [23–27]. The manufacture and secretion of IL-17 from Th17 cells appears to be dependent upon differentiation of naïve T cells into Th17 cells and the stimulation of these cells to secrete IL-17. Th17 cell differentiation depends upon the local milieu of cytokines. Indeed, several studies have implicated a cytokine concoction, consisting of at least IL-23, TGFβ, IL-6, IL-1β, and IL-21, that can induce differentiation of naïve T cells into Th17 cells [23–27]. Conversely, the presence of IFNγ, IL-12, and IL-4 inhibits Th17 differentiation and commits naïve T cells to either Th1 or Th2 cell lineages. While IL-23 appears to have a role in Th17 differentiation, IL-23 is thought to stimulate activated Th17 cells to release IL-17 [23, 25–28]. Proof of concept comes from a mouse model of spondylarthritis, where mice treated with anti-IL-23 antibody before the onset of disease, but not after, can be protected from developing spondylarthritis [29]. Despite this evidence, IL-23 inhibitors have largely proven ineffective in treating axSpA in human trials, suggesting other cytokines like IL36, IL-1β, IL-6, and IL-7 may have important roles in IL-17 production [30].

While the release of IL-17 from Th17 cells appears to be at least in part dependent on IL-23, IL-17 production in γ/δ T cells may also be IL-23 independent. γ/δ T cells are resident cells in the ciliary body, aortic root, colon, and enthesis [31–33]. Interestingly, these anatomical locations are commonly affected in axial spondyloarthritis and psoriatic arthritis. A portion of these cells are dependent on IL-23 [33]. However, other γ/δ T cells function independently of IL-23 and have been demonstrated to participate in physiological functions such as angiogenesis and gastric epithelial integrity [31]. The IL-23-independent and IL-23-dependent pathways may both be involved in the aetiology of SpA, which includes axial spondyloarthritis and psoriatic arthritis.

Due to the IL-23/IL-17 axis’s pro-inflammatory role, there is interest in its role in pathogenesis and as a potential treatment target. The IL-23/IL-17 axis has been implicated in several rodent models of disease (rheumatoid arthritis, inflammatory bowel disease, spondylarthritis, experimental autoimmune encephalitis, asthma) [24, 27, 34–37]. These cytokines are also elevated in serum and synovial fluid from patients with lupus, rheumatoid arthritis, and ankylosing spondylitis, [38–40]. The serum of patients with psoriasis and axSpA also shows increased levels of differentiated Th17 cells [41]. This axis has also been targeted clinically with varying success in spondylarthritis (reviewed by Siebert and colleagues [42••]). The most recent agent in this class of medications is bimekizumab, which targets IL-17A and IL-17F. Below, we will review the role of the IL-23/IL-17 axis in the pathology of AxSpA including ankylosing spondylitis and psoriatic arthritis with a particular focus on IL-17A and IL-17F.

## IL-17 in the Pathobiology of Ankylosing Spondylitis and Psoriatic Arthritis

Axial spondylarthritis, both radiographic and non-radiographic, is characterized by pain, stiffness, and limited range of motion of the axial skeleton and is associated with inflammation at the sacroiliac joint [43, 44]. Similarly, psoriatic arthritis is a related immune-mediated disease that affects the peripheral and axial skeleton, as well as the skin and nails. The IL-23/IL-17 axis appears to be involved in the pathogenesis of axial spondylarthritis and psoriatic arthritis. The levels of IL-17 producing cells are raised in the blood, enthesis, and synovial fluid in patients with spondylarthritis [39, 41, 45–47]. Single-nucleotide polymorphisms in the IL-23/IL-17 signalling axis are linked to ankylosing spondylitis and psoriatic arthritis [45, 48, 49]. Furthermore, targeting the IL-23/IL-17 signalling pathway with medication has shown improvement in patients with axSpA [42••, 45].

While genomewide associations have uncovered multiple proteins (including ERAP and IL-23 receptor) associated with axSpA, the strongest genetic association with ankylosing spondylitis and psoriatic arthritis is HLA-B27 [50, 51]. Interestingly, HLA-B27 may cause the presentation of arthritogenic peptides on the cell surface [52]. In addition, the HLA-B27 mutation delays the folding of the HLA protein, leading to an unfolded protein response [53]. The unfolded protein response promotes IL-23 secretion [54–56]. Therefore, patients with the HLA-B27 mutation may have increased inflammation at baseline, predisposing these patients to arthritis and other manifestations of autoimmune diseases.

Another theory (the joint-gut axis theory) posits that the increased inflammation in axSpA may begin in the intestinal tract [57]. Ankylosing spondylitis and psoriatic arthritis are associated with inflammatory bowel disease. It has been shown that axSpA patients without overt inflammatory bowel disease are found to have subclinical inflammatory changes in the bowel [58, 59]. For example, the intestinal lamina propria of ankylosing spondylitis has increased levels of mononuclear cells [60]. IL-23, but not IL-17A, levels are increased in intestinal biopsies of patients with ankylosing spondylitis [61]. This inflammation can lead to translocation of bacteria and bacterial products across the intestinal wall into the blood, which can be reversed with antibiotics [62]. The IL-23 released from cells activated in the intestines is thought to circulate and lead to the release of IL-17 [11••, 63, 64]. Indeed, in the HLA-B27 transgenic mouse model for ankylosing spondylitis, animals do not develop ankylosing spondylitis when raised in a germ-free environment [65]. Innate natural killer T cells and mucosal activated invariant T cells activated by mucosal inflammation can produce IL-17, and these cells can be found in the peripheral blood and synovial fluid [66, 67]. Therefore, IL-23 and IL-17 derived from intestinal inflammation may lead to the joint inflammation in axSpA.

As discussed above, IL-17 may be released in an IL-23-dependent or IL-23-independent manner. Clinical trials in blocking IL-23 signalling in patients with ankylosing spondylitis have proven to be ineffective [30]. One possibility is that cells responsible for releasing IL-17 have been differentiated and are in situ by the time therapy has begun. van Tok and colleagues demonstrated this in a mouse model of ankylosing spondylitis. Mice over-expressing human HLA-B27 developed arthritis if IL-23 antibodies were given after arthritis developed, but not if these antibodies were injected prophylactically [29]. Indeed, resident entheseal T cells were found to release IL-17 in an IL-23-dependent manner [33]. Interestingly, Cuthbert and colleagues have also demonstrated a population of T cells that release IL-17 independently of IL-23 [32].

Taken together, these lines of evidence suggest there is an environmental trigger that leads to an imbalance in the cytokine milieu in the joint leading to inflammation and potentially overzealous repair.

## Targeting IL-17 in Ankylosing Spondylitis and Psoriatic Arthritis

As IL-17 is central to the pathogenesis of axSpA, several biological agents have been developed against IL-17 or its target receptors [68, 69]. The biologics and their targets are summarized in Table 2.

**Table 2:** IL-17 therapeutics and their targets

Therapeutic	Target
Secukinumab	IL-17A
Ixekizumab	IL-17A
Bimekizumab	IL-17A/17F
Brodalumab	IL-17RA

In randomized control trials, secukinumab was shown to be effective in radiographic axSpA (ankylosing spondylitis) on multiple measures of disease activity (ASAS20, ASAS40, BASDAI, ASDAS) and these improvements were durable for 5 years on extension of these trials [70–75]. Encouragingly, improvements in disease control were also noted in patients who failed previous TNF-α inhibitor treatment [72].

Without treatment, the erosion and inflammation of joints in ankylosing spondylitis can lead to pain and decreased mobility. Several clinical trials have demonstrated that secukinumab prevents or reduces the progression of radiographic change over 2 years [75–77]. Secukinumab also can decrease inflammation that can only be detected on MRI [70].

Ankylosing spondylitis can negatively impact the patient’s quality of life [78, 79]. In addition to objective measures of disease control, there are various patient-reported outcomes (PROs) that measure subjective improvements in the disease process. In these studies, patients answer surveys that address their pain, fatigue, mood/mental health, general health, sleep, and impact on activities of daily living. Post hoc analysis of secukinumab randomized clinical trials showed that secukinumab improved mental and physical health, as well as their perceived quality of life [80•, 81–83]. In addition to their improved quality of life, these patients reported less work absenteeism and increased work productivity [81]. Patients treated with secukinumab also reported less fatigue [81, 84]. The above reported outcomes were noted after 16 weeks of treatment and persisted to the end of the follow-up period (52 weeks).

Secukinumab was well tolerated, with the most common side effects being nasopharyngitis, headache, diarrhoea, and upper respiratory tract infection (URTI). There was only one severe infection for every 100 patient years [70, 71, 73]. Braun and colleagues showed similar findings for secukinumab in a 4-year trial where the most common adverse effect was viral URTIs and there was only one case of tuberculosis [74].

Secukinumab was also effective in psoriatic arthritis. Through 16 weeks of treatment, Mease and colleagues found that patients treated with secukinumab had significant improvements across musculoskeletal domains and striking improvements in psoriasis, with PASI 100 scores of up to 40–50% [85]. Moreover, these improvements were maintained for 2 years of treatment [86, 87]. Similar results were found in trials with various doses and delivery methods [88–91]. One of the cardinal features of psoriatic arthritis is tendon inflammation which manifests as enthesitis and dactylitis. Secukinumab treatment results in resolution of enthesitis and dactylitis in 40–60% of patients who had these domains involved at baseline [87, 88, 90, 91]. Secukinumab treatment also results in minimal radiographic progression in affected joints up to 3 years [87, 91–93]. This was also reflected in MRI imaging showing decreased joint inflammation [85]. As with ankylosing spondylitis, patient-reported outcomes are also important for psoriatic arthritis. A post hoc analysis of randomized controlled trials showed that secukinumab improved patient-reported outcomes on multiple facts including quality of life, fatigue, global assessment of disease, and overall health status [94, 95]. Again, secukinumab was well tolerated with few severe side effects and was also effective in patients that had failed previous treatment with TNF-α inhibitors [96].

The EXCEED trial has compared secukinumab to adalimumab monotherapy in a head-to-head trial over 52 weeks in patients with psoriatic arthritis [97•]. The primary outcome was the American College of Rheumatology (ACR) 20 response, which showed a higher percentage of responders with secukinumab. However, this was not significantly increased as compared to adalimumab using intention to treat analysis. This did not reach significance if the stricter non-responder imputation was used. Although secukinumab was not significantly better than adalimumab based on musculoskeletal measures, secukinumab was associated with better skin responses as measured by the PASI score. There were no differences in adverse effects and tolerability between these medications. This may steer providers to treat patients with secukinumab if there is significant skin involvement.

Similarly, ixekizumab was designed to target IL-17A as well. In a randomized control trial (COAST-V) of nearly 400 biological DMARD-naïve radiographic axial spondyloarthritis patients, ixekizumab had greater proportion of patients achieving ASAS40 within the first 2 weeks of the trial; this plateaued at around 50% of patients in the trial [98]. Furthermore, there were improvements on other measures of disease activity as well as decreased biochemical and MRI signs of inflammation [98]. This trial included an active comparator arm of patients treated with adalimumab that performed consistently with previous trials with adalimumab. Although the trial was not powered to compare head to head with adalimumab with ixekizumab, ixekizumab was non-inferior to adalimumab. A similar trial (COAST-W) was carried out in patients with previous inadequate response to TNF-α inhibitors. Patients enrolled in this trial showed similar results to COAST-V after 16 weeks of treatment [99]. After 16 weeks, the participants of the COAST-V and COAST-W trials were pooled and followed for a 2-year extension trial. The results after 52 weeks showed that the initial improvements noted above were maintained. Post hoc analysis of the COAST-X, COAST-W, and COAST-V trials shows that patient-reported outcomes relating to quality of life, fatigue, function, health status, and work productivity improved with ixekizumab [100–103]. Interestingly, these improvements in patient-reported outcomes were correlated with the patients’ ASAS20/40 response [100, 102]. Further analysis of the clinical trial data showed that 75% of patient had no radiographic progression over 2 years [104]. In addition, of patients with available MRI data, 96% had minimal worsening of inflammation [104]. Furthermore, ixekizumab was well tolerated with the most common side effect being nasopharyngitis. Impressively, 88% of patients remained on the study medication at the end of 52 weeks [105]. In addition, ixekizumab was also shown to be effective and well tolerated in a trial with non-radiographic axSpA [106].

As with secukinumab, ixekizumab was also trialled in patients with psoriatic arthritis. There was improvement in joint disease, dactylitis, and skin disease [85, 96, 107, 108]. These improvements were also reflected with minimal radiographic changes even up to 3 years of treatment [109, 110]. There was a similar improvement in patient-reported outcomes [111–113]. Long-term follow-up of both medications showed that these medications had long-term efficacy in psoriatic arthritis and were well tolerated [86, 96, 110]. The side effects were consistent with those found in ankylosing spondylitis patients treated with these medications [86, 96, 114].

As with secukinumab, ixekizumab was directly compared with adalimumab in patients with psoriatic arthritis in the open-label SPIRIT-H2H trial [115, 116•]. Unlike EXCEED, SPIRIT-H2H compared patients who were on biological medications alone or concomitantly with methotrexate. The SPIRIT-H2H primary outcome was a composite of PASI 100 and ACR 50. Ixekizumab was significantly better beginning in week 8 in patients only on biological medications. This advantage was held through to the end of the study (52 weeks). While improvements in the primary outcome were driven primarily by PASI 100, a further analysis of the same subgroup of patients (biological medications only) showed that ixekizumab also improved on some musculoskeletal outcomes. Ixekizumab had a better ACR 70 response as compared to adalimumab. Furthermore, as per the Disease Activity in Psoriatic Arthritis (DAPSA) score, significantly more patients treated with ixekizumab had mild or very low disease. In addition, patients with ixekizumab performed better on the Health Assessment Questionnaire Disability Index (HAQ-DI), which measures patient performance on activities of daily living. However, the advantage described above for ixekizumab was seen when methotrexate was used in combination with the biological medications. These findings suggest that ixekizumab may outperform adalimumab if used as first-line monotherapy in psoriatic arthritis.

While secukinumab and ixekizumab block signalling through IL-17A, it does not affect the signalling through the other family members in the IL-17 cytokine family. As described previously, IL-17A and IL-17F can form a heterodimer that signals through a receptor consisting of at least IL-17RA and IL-17RC [7]. As with IL-17A, IL-17F is released from activated T cells and monocytes [9] and has been shown to be involved inflammation in in vitro and mouse models of disease [5, 117]. Furthermore, IL-17A, IL-17F, and their receptors are found in the inflamed synovium of patients with rheumatoid arthritis and psoriatic arthritis [118].

Brodalumab was the first biologic that targeted other IL-17 family members in addition to IL-17A by targeting the IL-17 receptor complex IL-17RA/IL-17C and preventing its activation [119]. This medication was first trialled in psoriasis patients; however, these trials reported an increase in psychiatric adverse events, including depression, anxiety, and suicidal ideation. In the sponsor product information, there was a clear signal for suicidal ideation and behaviour. In 6243 patients for a total of 10,438 patient years, there were 39 patients with suicidal ideation or behaviour events (0.37/100 patient years). Of these events, 18 patients had suicidal behaviour and 6 patients had completed suicide [120].

However, meta-analysis of over 4000 patients treated with brodalumab for 5 years found there was no increase in psychiatric adverse events as compared to other psoriasis trials [121]. In axSpA, brodalumab was shown to be effective in treating the musculoskeletal symptoms in as early as 2 weeks [122•, 123, 124•]. In these short trials, brodalumab was well tolerated with the most common side effects being infection. Reassuringly, there was no increase in psychiatric adverse effects [122•, 123, 124•].

With all biological medications targeting the Il-17 pathway, candidia infections and the development of inflammatory bowel disease (Crohn’s disease and ulcerative colitis) are a concern. Candida infections are a specific concern because Il-17 is involved in fungal defence. In axSpA patients treated with secukinumab, the levels of candida infections were low (0.1–3.2 events/100 patient years [72–75, 87, 89, 90, 93]. Similarly, axSpA patients treated with ixekizumab and brodalumab had low levels of candida infection [98, 105, 106, 125]. A meta-analysis of randomized control trials of IL-17 inhibitors in psoriasis and psoriatic arthritis showed that only 0–5% of patients developed candida infections [126]. Encouragingly, these candida infections were mild to moderate and resolved with topical or oral treatment and did not result in discontinuation of the study medication [126]. The findings from this meta-analysis are in keeping with the trials studied here.

As described in previous sections, IL-17 is involved in repair of mucosal surfaces. Therefore, IL-17 inhibition may worsen or lead to the development of IBD [127]. As with candida infections, IBD is a rare side effect of IL-17 blockade. A meta-analysis of randomized control trials of IL-17 blockade in psoriasis and axSpA found there was no increase in IBD incidence as compared to placebo, and this risk was not increased over 2 years of treatment [128]. A real-world observational study of the French National Health database found that there was no increased risk with IL-17 inhibitor treatment when these patients were compared to patients treated with etanercept [129•].

The newest agent targeting the IL-17 pathway is bimekizumab, which was initially tested in psoriasis, but phase II trials have found it to be effective in both radiographic and non-radiographic axSpA. Bimekizumab targets both IL-17A and IL-17F; however, unlike brodalumab, it targets the cytokines directly as opposed to the receptor. In the BE AGILE trial, more than 300 patients were randomized to placebo or different doses of bimekizumab and their clinical improvement was measured by ASAS40. By the end of the 12 months, nearly over 40% of patients treated with 64 mg or more of bimekizumab showed improvement as per ASAS40. In addition to improvements based on ASAS40, there was an improvement in sacroiliac and spinal inflammation on MRI imaging. There were also improvements in patient-reported outcomes. Furthermore, these improvements were maintained for 48 weeks after re-randomization of all patients to either 160 mg or 320 mg [130]. The medication was well tolerated, with the most common side effects being nasopharyngitis, pharyngitis, bronchitis, upper respiratory infections, and oral candidiasis. For oral candidiasis, there were 7.5 events/100 patient years. These mediations were mild to moderate and resolved with topical or oral treatment. Specifically, for IBD, the rate was 0.77 exposure-adjusted incidence/100 patient years for Crohn’s and ulcerative colitis. Indeed, two out of the four total IBD cases were exacerbation of previous IBD and two were new diagnoses. Similarly, the BE ACTIVE trial was a phase IIb trial that randomly assigned patients with psoriatic arthritis to placebo or a dose of bimekizumab. Again, the results were measured at 12 weeks and the patients were re-randomized to receive either 160 mg or 320 mg for a total of 48 weeks of the medication. As with the BE AGILE trial, the patients in BE ACTIVE showed improvements in their joint function as per the ACR 50 and their skin disease as per the PASI scores. Furthermore, a greater number of bimekizumab-treated patients had enthesitis resolution as compared to placebo-treated ones. In addition to musculoskeletal and skin improvements, patients also had measurable improvements in quality of life and reduced disability with bimekizumab treatment. Furthermore, the medication was again well tolerated with nasopharyngitis, pharyngitis, bronchitis, upper respiratory infections, and oral candidiasis being the most common side effects. There were 5% of patients that developed oral candidiasis, all of which resolved with topical or oral treatment. These infections did not result in discontinuation of bimekizumab. There were no cases of IBD with bimekizumab treatment in psoriatic arthritis patients. There were few patients who ceased the trial medication due to side effects [131]

Although further research is required, these early results are promising. Bimekizumab trials in psoriasis (BE VIVID, BE READY, BE RADIANT, BE SURE) suggest that it may be more efficacious than ustekinumab (IL-12/IL-23 inhibitor), secukinumab, or adalimumab [132–136]. Unfortunately, similar comparisons have not been made for bimekizumab in psoriatic arthritis and ankylosing spondylitis. However, as the pathology of psoriasis still depends in part on IL-17, this is a promising early sign that bimekizumab may be more effective than previous treatment. Encouragingly, a recent press prelease of BE MOBILE 1 and BE MOBILE 2 studying bimekizumab in non-radiographic and radiographic axSpA showed that patients randomized to bimekizumab had positive responses as per ASAS40 and all secondary end points (i.e. BASDAI, ASDAS). Furthermore, bimekizumab was well tolerated in these patients with the safety profile in line with previous studies [137, 138].

## Conclusion

The development of biologics targeting the IL-17 pathway has been exciting as they have opened a novel therapeutic target. Furthermore, these medications are exciting as they have shown to be efficacious even in patients that have failed therapies targeting TNF-α. Bimekizumab is the newest therapy targeting this pathway. The studies suggest that this therapy may be more effective than previous therapies if the studies in psoriasis can be extrapolated to ankylosing spondylitis and psoriatic arthritis. We await the publication of phase 3 trials of bimekizumab for further data elucidating the efficacy of this therapy.
